# Gender Matters in the Receipt of Informal Care in Later Life: A Cross-National Comparison Across the USA, Korea, and China

**DOI:** 10.1093/geroni/igaa057.1845

**Published:** 2020-12-16

**Authors:** Minyoung Kwak, BoRin Kim, Hyunjoo Lee, Jiaan Zhang

**Affiliations:** 1 Daegu University, Gyeongsan, Republic of Korea; 2 University of New Hampshire, Durham, New Hampshire, United States

## Abstract

This study compares patterns of gender difference in the receipt of informal care among community-dwelling older adults across the United States, Korea, and China. Data came from the 2014 HRS, the 2014 KLoSA, and the 2015 CHARLS. Logistic regression models were used to predict the receipt of informal care by gender. We also examined how the effects of health and living arrangement on the receipt of informal care differ depending on gender. In the United States and China, older women were more likely to receive informal care than men. However, older Korean women were less likely to receive informal care than men. The effects of health and living arrangement on the use of informal care were moderated by gender in different ways across countries. Discussions include implications for practice and policy to reduce the gender gap in the receipt of informal care.

